# A retrospective study for the diagnostic value of chromosomal microarray analysis in fetuses with high-risk prenatal indications

**DOI:** 10.3389/fgene.2025.1649253

**Published:** 2025-08-25

**Authors:** Hui Xiao, Junfang Xiao, Huan Zhang, Shuhui Huang, Qing Lu, Huizhen Yuan, Yongyi Zou, Bicheng Yang, Yanqiu Liu

**Affiliations:** Department of Medical Genetics, Jiangxi Maternal and Child Health Hospital, Nanchang, China

**Keywords:** chromosomal microarray analysis, copy number variations, prenatal diagnosis, genetic counseling, high-risk prenatal indications

## Abstract

**Objective:**

The aim of this study was to determine the diagnostic value of prenatal chromosomal microarray analysis (CMA) for fetuses at high risk for various conditions on chromosomal abnormalities.

**Methods:**

In the study, 8,560 clinical samples were collected from pregnant women between February 2018 and June 2022, including 75 villus, 7,642 amniotic fluid, and 843 umbilical cord blood samples. All samples were screening for chromosomal abnormalities using both CMA and karyotyping. This retrospective analysis included 8,560 pregnancies with high-risk indications for invasive prenatal diagnosis, mainly including ultrasound anomalies, high risk for maternal serum screening (MMS), high risk for non-invasive prenatal tests (NIPTs), family history of genetic disorders or birth defects, and advanced maternal age (AMA). All samples were evaluated using invasive CMA. The rate of clinically significant genomic imbalances between the different groups was compared.

**Results:**

The success rate of CMA was 99.95% (8,556/8,560). A total of 1,037 samples (12.11%, 1,037/8,560) were presented with chromosomal abnormalities using CMA, whereas 803 samples (9.38%, 803/8,560) were shown with chromosomal abnormalities using karyotyping. The overall prenatal diagnostic yield was 1,040 (12.14%) of 8,560 pregnancies. Clinically significant genomic aberrations were identified in 153 (6.21%) of 2,463 patients with non-structural ultrasound anomalies, 79 (6.38%) of 1,238 with structural ultrasound anomalies, 37 (4.26%) of 868 at high risk from MSS, 395 (42.29%) of 934 at high risk from NIPTs, 16 (2.94%) of 544 with a family history, 7 (1.89%) of 369 with AMA, 1 (1.56%) of 64 with a history of adverse exposure, 10 (4.46%) of 224 with parental chromosome anomaly, and 9 (2.99%) of 301 with other indications.

**Conclusion:**

CMA has a greater diagnostic value for screening chromosomal abnormalities, especially in pregnant women with normal karyotypes. The diagnostic yields of CMA for pregnancies with different indications greatly varied. CMA could serve as a first-tier test for structural anomalies, especially multiple anomalies, hydrops fetalis, cystic hygroma, and thickened nuchal translucency or nuchal fold.

## 1 Introduction

Chromosomal microarray analysis (CMA) enables genome‐wide detection of submicroscopic chromosomal imbalance or copy number variation. The CMA was widely used to detect a variety of abnormalities, including chromosome aneuploidies, unbalanced rearrangements, microdeletions and microduplications, triploidy, uniparental isodisomy, and low‐level mosaicism, which are not routinely detected using karyotyping. Karyotyping has long been established as the “gold standard” in the evaluation of developmental disorders in children (Michelson et al., 2011).

Studies have established the superior yield of CMA over standard karyotypes in the prenatal detection of clinically relevant chromosomal abnormalities. CMA can reveal an additional molecular diagnosis in approximately 6%–10% cases than karyotyping in fetuses with structural anomalies (Hillman et al., 2013; Callaway et al., 2013; Wapner et al., 2012). Following the application in prenatal diagnosis, the American College of Obstetricians and Gynecologists (ACOG) and the Society for Maternal‐Fetal Medicine (SMFM) published joint recommendations regarding the use of micro-array analysis in prenatal diagnosis. Following these recommendations, considerable experiences and mass of data have been accumulated from detection of more kinds of chromosomal abnormalities using CMA. Karyotype analysis is an established technique, whereas CMA is a relatively new molecular diagnostic technology. The time required for diagnosis by karyotype analysis is longer than that for CMA because of the amniotic fluid cell culture, whereas CMA can use DNA directly. CMA can detect micro-deletions and micro-duplications of chromosomes but cannot detect balanced structural abnormalities, such as balanced translocation and inversion of chromosomes. Therefore, the two diagnostic methods for prenatal chromosome analysis are widely combined in recent years (Cicatiello et al., 2019; Vo et al., 2024; Xue et al., 2024).

Accompanied by challenges, more results like variants of uncertain significance (VUS) were identified, which are not clearly associated with known syndromes but may act as risk factors, and have become a primary concern for patients and genetic counselors (Yin et al., 2025; Richards et al., 2015). Meanwhile, significant advances were also made in prenatal screening technologies, such as non-invasive prenatal test (NIPT) and ultrasonic imaging. Therefore, it is important to reassess current trends and the clinical utility of CMA to provide reference values for prenatal counseling.

In this retrospective study, we aimed to investigate the potential of CMA for screening chromosomal abnormalities in prenatal diagnosis and applying CMA to fetuses with different indications to provide reference values for clinicians.

## 2 Materials and methods

### 2.1 Participants and samples

Prenatal samples (n = 8,560) of villus, amniotic fluid, or fetal umbilical cords were received at our laboratory between 2018 and 2022 for genetic diagnoses using microarrays. Prenatal invasive CMA was conducted at our prenatal center for fetuses with high-risk indications, including abnormal ultrasound findings, maternal serum screening, NIPT, family history of genetic disorders or birth defects, advanced maternal age, and other indications. Karyotyping results were available for 8,498 of the 8,560 prenatal samples. All the pregnant women provided informed parental consent before genetic testing. The study was approved by the Maternal and Child Health Hospital of Jiangxi province. Fetal ultrasound anomalies in this study included non-structural and structural types. Fetuses with non-structural anomalies were nonspecific anomalies basically assigned to three subgroups, namely, ultrasound soft markers (USMs), abnormal amniotic fluid volume, and fetal growth restriction (FGR). Fetuses with structural anomalies were assigned to ten subgroups, cardiovascular, urogenital, musculoskeletal, abdominal, hydrops fetalis, cystic hygroma, central nervous, craniofacial, thorax dysplasia and dysplasia of the other organs.

The cut-off risks of maternal serum screening for Down syndrome and Edwards syndrome are 1/270 and 1/350, respectively, and 1/1,000 is the cut-off risk for critical risk.

High risk for NIPT indicated potential aneuploidies or gross chromosomal aberrations based on cell-free DNA in maternal plasma. Chromosome aneuploidy risk was evaluated using a Z-score ratio (|Z| ≥ 3 indicates a high risk of chromosomal aneuploidies, and |Z| < 3 indicates a low risk) (Liang et al., 2019).

Family history in this study refers to abnormal pregnancy with genetic disorders, chromosomal abnormalities, and other unexplained abnormal pregnancy–labor history.

Parental chromosome anomaly refers to one partner’s lymphocyte chromosome abnormality, including balanced or Robertsonian translocation, inversion, and other aberrations that may increase the risk of chromosomal abnormalities in the offspring.

History of adverse exposure during pregnancy refers to women using drugs that may harm the fetus or being exposed to ionizing radiation during pregnancy.

Advanced maternal age refers to pregnant women over the age of 35 years at delivery.

### 2.2 CMA platform and copy number variation (CNV) classification

Fetal genomic DNA was extracted from villus, amniotic fluid, and fetal cord blood. We also extracted DNA from maternal peripheral blood samples to exclude maternal contamination. All prenatal samples were tested using Affymetrix CytoScan 750K arrays (Affymetrix, Santa Clara, CA, United States), as described by the manufacturer. The copy number variations (CNVs) were interpreted according to appropriate databases of pathogenic and benign variants, including Online Mendelian Inheritance in Man (OMIM, http://omim.org/), the Database of Chromosomal Imbalance and Phenotype in Humans Using Ensembl Resource (DECIPHER, http://decipher.sanger.ac.uk/), the Database of Genomic Variants (DGV, http://projects.tcag.ca/variation), and ClinVar (http://www.ncbi.nlm.nih.gov/clinvar/).

According to the American College of Medical Genetics (ACMG) practice guidelines, each CNV was assigned to one of the five categories of clinical significance as follows: pathogenic, likely pathogenic, VUS, likely benign, and benign (Riggs et al., 2020).

### 2.3 Statistical analysis

Statistical analyses were performed using SPSS software v22.0 (SPSS Inc., Chicago, IL, United States). The chi-square test and two independent-sample t-tests were applied to analyze the data. Differences were considered statistically significant when *P* < 0.05. The multiple-comparison correction method used in this study is the Bonferroni correction.

## 3 Results

### 3.1 Diagnostic efficacy of prenatal CMA

We retrospectively analyzed 8,560 prenatal samples with clinical indications obtained at 11–33 weeks of gestation, including 75 villus (0.87%, 75/8,560), 7,642 amniotic fluid (89.27%, 7,643/8,560), and 843 umbilical cord blood (9.84%, 843/8,560). The clinical indications of this cohort were as follows: ultrasound anomalies (n = 3,701, 43.23%), including non-structural ultrasound anomalies (n = 2,463, 28.77%) and structural ultrasound anomalies (n = 1,238, 14.46%), risk for maternal serum markers (n = 987, 11.52%), positive non-invasive prenatal test results (n = 943, 11.02%), family history of genetic disorders or birth defects (n = 544, 6.35%), parental chromosome anomaly (n = 223, 2.6%), history of adverse exposure during pregnancy (n = 64, 0.74%), advanced maternal age (n = 369, 4.31%), two kinds of abnormal indications (n = 1400, 16.35%), three kinds of abnormal indications (n = 45, 0.52%), and other indications (n = 301, 3.51%). Table 1 shows the diagnostic yield for different indications.

**TABLE 1:** Analysis of the relationship between chromosomal abnormalities and clinical indications.

Variable	Number of cases	Pathogenic CNVs		DR (%)
		>10 Mb	<10 Mb	
Advanced maternal age	369	5	2	1.89
Abnormal ultrasound	3,701	134	98	6.26
Risk of serological screening	987	21	20	4.15
High risk of NIPT	934	356	39	42.29
Adverse family history	535	4	12	2.94
Parental chromosome anomaly	224	5	5	4.46
History of adverse exposure	64	1	0	1.56
Two kinds of abnormal indications	1,400	284	29	22.35
Three kinds of abnormal indications	45	10	3	28.88
Others	301	2	7	2.99

**FIGURE 1:**
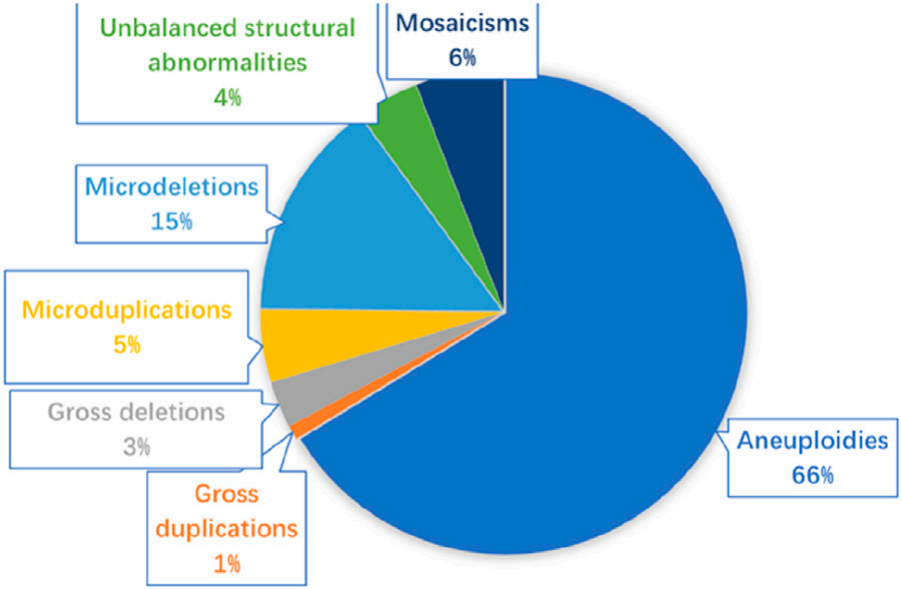
Various types of genetic anomalies identified in 8,560 fetuses. Micro-duplication/micro-deletion: segmental genomic imbalances <10 Mb; gross duplication/gross deletion: segmental genomic imbalances >10 Mb.

In the 8,560 samples, 62 cases of samples were not successfully detected with karyotyping, including 10 villus and 52 amniotic fluid, and the success rate of karyotyping was 99.27% (8,498/8,560). A total of 803 samples (9.38%, 803/8,560) were found to exist chromosomal abnormalities using karyotyping, including 676 cases of numerical abnormalities (84.18%, 676/803), 78 cases of structural abnormalities (9.71%, 78/803), and 49 cases of chimeras (6.1%, 49/803). In the 8,560 samples, the success rate of CMA was 99.95% (8,556/8,560). A total of 1,463 (17.09%, 1,463/8,560) samples were found to have chromosomal alterations. Among them, 1,037 clinically significant genomic alterations were identified, including 686 cases of aneuploidies (66.15%, 686/1,037), 68 cases of large-fragment abnormality (≥10 Mb) (6.55%, 68/1,037), 218 cases of small-fragment abnormality (<10 Mb) (21.02%, 218/1,037), 61 mosaicisms (5.88%, 61/1,037), and 3 cases of absence of heterozygosity (AOH) (0.28%, 3/1,037). Gross duplications or deletions were CNVs >10 Mb, which usually can be identified using karyotyping, whereas micro-duplications or micro-deletions were CNVs <10 Mb, which generally only be detected using CMA. The 68 cases of large-fragment abnormality included 32 cases of deletion (47.05%, 32/68), 10 cases of duplication (14.7%, 10/68), and 27 cases of deletion and duplication (39.7%, 27/68). The 218 cases of small fragment abnormality included 153 cases of micro-deletion (70.18%, 153/218), 49 cases of micro-duplication (22.47%, 49/218), and 16 cases of deletion and duplication (7.33%, 16/218). In addition, 227 (2.94%, 227/7,695) samples with normal karyotypes were found to have chromosomal abnormalities. Figure 1 shows the various types of genetic anomalies identified.

### 3.2 CMA in fetuses with high-risk prenatal indications

#### 3.2.1 CMA in fetuses with ultrasound anomalies

The association between fetal ultrasound anomalies and chromosomal abnormalities has already been recognized, such as Down syndrome (trisomy 21), Edwards syndrome (trisomy 18), and Patau syndrome (trisomy 13). Micro-deletions and micro-duplications that involve clinically significant genomic regions are also associated with specific genetic syndromes, which may show congenital abnormal phenotypes. For example, approximately 1/100 of fetuses with congenital heart disease are associated with DiGeorge syndrome, which is caused by a micro-deletion of the region of chromosome 22 (22q11.2 deletion syndrome) (McDonald-McGinn et al., 2015).

Clinically significant CNVs were detected in 232 of the 3,701 fetuses with abnormal ultrasound findings. The genomic imbalances of these 232 fetuses included aneuploidies (n = 102), gross deletions (n = 7), gross duplication (n = 5), unbalanced translocations (n = 16), micro-deletions (n = 69), micro-duplication (n = 19), and mosaicisms (n = 14). Fetal ultrasound anomalies in this study included non-structural and structural types. Table 2 shows the diagnostic yield for each subgroup of ultrasound abnormalities.

**TABLE 2:** Diagnostic yield for specific indications of fetal ultrasound anomalies.

Ultrasound category	Number of cases	Pathogenic CNVs		DR (%)
		>10 Mb	<10 Mb	
Non-structural anomalies	2,463	91	62	6.21
USM	2,351	89	57	6.21
FGR	37	0	1	2.70
Polyhydramnios/oligohydramnios	24	0	0	0
Two or three anomalies	51	2	4	11.76
Structure anomalies	1,238	43	36	6.38
Isolated in the single system	1,025	29	27	5.46
Multiple system anomalies	90	8	6	15.50
Structure anomalies combined with non-structural anomalies	123	6	3	7.31

USM, ultrasonographic soft markers; FGR, fetal growth restriction.

The non-structural ultrasound anomalies (n = 2,463) are more common than structural ultrasound anomalies (n = 1,238) in the fetus, and the chromosomal abnormality detection ratio is higher for structural (6.38%,79/1,238) than for non-structural (6.21%,153/2,463) types. Table 3 shows the diagnostic yield for each subgroup of ultrasound abnormalities.

**TABLE 3:** Detection rate of fetal ultrasound soft marker distributions.

USM	Number of cases	Pathogenic CNVs		DR (%)
		>10 Mb	<10 Mb	
Multiple soft markers	366	12	9	5.73
EICF	240	7	5	5.0
Ventriculomegaly	133	1	3	3.0
Enlarged cisterna magna	21	1	0	4.76
CPCs	142	0	2	1.4
Increased NT/NF	640	61	16	12.03
Shortened long bone	13	0	0	0
Echogenic bowel	36	0	0	0
Mild hydronephrosis	44	0	1	2.27
Absent/hypoplastic nasal bone	284	5	3	2.81
ARSA/RAA	174	1	9	5.74
SUA	95	0	4	4.21
PLSVC	60	1	1	3.33
PRUV	39	0	1	2.56

EICF, echogenic intracardiac focus; CPCs, choroid plexus cysts; ARSA, aberrant right subclavian artery; RAA, right aortic arch; SUA, single umbilical artery; PLSVC, persistent left superior vena cava; PRUV, persistent right umbilical vein.

##### 3.2.1.1 Non-structural ultrasound abnormalities

The prevalence of fetuses with USMs was the most among the non-structural anomalies, and the incidence of clinically significant genomic alterations was 6.21% (146/2,351), which was higher than that of other isolated non-structural abnormalities. USMs refer to sonographic findings that are nonspecific, often transient, and can be readily detected using prenatal ultrasonography (Van den Hof et al., 2005). Although USMs are commonly distinct from fetal structural malformations or FGR, they might indicate chromosome abnormal risks (Breathnach et al., 2007). The USMs mainly included echogenic intracardiac focus (EICF), ventriculomegaly, enlarged cisterna magna, choroid plexus cysts (CPCs), thickened nuchal translucency or nuchal fold (NT/NF), echogenic bowel, mild hydronephrosis, shortened long bones, aberrant right subclavian artery (ARSA), absent or hypoplastic nasal bone, and single umbilical artery (SUA).

Fetuses can have single or multiple USMs. Among the 2,351 fetuses with USM, 1,985 and 366 had single and multiple USMs, respectively. The incidence of clinically significant genomic alterations of fetuses with multiple USMs is 5.73% (21/366), which was higher than most single USMs, except NT/NF(12.03%,77/640) and ARSA/RAA (5.74%, 10/174) subgroups (Table 3). Among the 37 FGR fetuses, only one pathogenic micro-duplication and deletion in 5p15.33p15.1 and 13q32.3q34 was detected, indicating chromosomal unbalanced translocation. In terms of abnormal AF volume, none of the 24 fetuses had clinically significant genomic alterations.

##### 3.2.1.2 Structural ultrasound abnormalities

Among the 1,238 fetuses with structural anomalies, isolated defects in the single system were the most frequent, with a clinically significant genomic alteration detection ratio of 5.43% (56/1,030). Multiple abnormalities were defects of two or more systems, and the group most closely related to chromosomal alteration was the multiple system anomalies group, with a detection ratio of 15.9% (14/88), the detection rate for which was obviously higher than that for single-system structural anomalies. Detection rates of different subcategories are shown in Table 4.

**TABLE 4:** Detection rate of fetal structural defect distributions.

Structural defect distributions	Number of cases	Pathogenic CNVs		DR (%)
		>10 Mb	<10 Mb	
Cardiovascular	214	7	8	7.0
Urogenital	244	3	6	3.68
Musculoskeletal	116	1	3	3.44
Abdominal	104	5	1	5.76
Hydrops fetalis	45	7	0	15.55
Cystic hygroma	48	4	2	12.5
Central nervous	73	2	4	8.21
Craniofacial	71	0	3	4.22
Thorax dysplasia	69	0	0	0
other	41	0	0	0

Among the subgroups of isolated structural anomalies, both urogenital (23.80%, 244/1,025) and cardiovascular (20.87%, 214/1,025) defects were the most frequent, whereas the pathogenic CNV detection ratio in the cardiovascular subgroup is relatively higher (7.0%,15/214) than that in the urogenital subgroup (3.68%,9/244). Cardiovascular defects varied, including simple ones like ventricular septal defect and complex ones like hypoplastic left heart syndrome or tetralogy of Fallot (TOF). Urogenital defects included polycystic kidney, multicystic dysplastic kidney, double collecting renal system, renal agenesis, and horseshoe kidney. The occurrence rate is followed by musculoskeletal abnormalities (11.31%, 116/1,025), mainly including clubfoot, vertebral malformation, curved extremities, and rib deformity, while the detection rate (DR) of pathogenic CNVs is 3.44%.

Abdominal defects included esophageal/intestinal atresia, intestinal dilatation, omphalocele, gallbladder abnormalities, and abdominal masses. Thorax dysplasia included diaphragmatic hernia, pulmonary cystadenoma, and pulmonary sequestration, whereas no pathogenic CNVs were identified in this subgroup. Craniofacial dysplasia included cleft lip and palate, face malformation, and auricular deformities. Central nervous abnormalities included agenesis–hypoplasia of the corpus callosum, hydrocephaly, holoprosencephaly, cerebellar anomalies, encephalocele, and microcephalus. Cystic hygroma mainly refers to neck hygroma and some lymphangioma found in the chest or craniofacial region. The highest pathogenic CNV DR was found in hydrops fetalis at 15.55% (7/45), including five cases of trisomy 21 and two cases of 45,X.

A total of 36 additional small pathogenic CNVs were detected using CMA in structure anomalies, increasing the DR by 2.9%. In addition, 62 additional small pathogenic CNVs were detected using CMA in non-structural anomalies, increasing the DR by 2.51%.

#### 3.2.2 CMA in fetuses with the risk of serological screening

There were a total of 987 fetuses identified with the risk of serological screening. Among 868 women at high risk of having a child with Down syndrome or Edwards syndrome, trisomy 21 (n = 6), trisomy 18 (n = 6), and 6 mosaicisms were detected. In addition, 19 additional small pathogenic CNVs were detected using CMA. The DR of pCNV in the high-risk subgroup is 4.26% (37/868). Among 101 women identified as critical risk, one 45,X/46,XY mosaicism, two 22q11.21 micro-duplications, and one 16p11.2 microdeletion with 46.8% penetrance were detected. As for the 18 cases with a single biochemical criterion MoM abnormality, no CNVs were identified. Diagnostic yields for the subcategorical risk of serological screening are shown in Table 5.

**TABLE 5:** Diagnostic yield for specific indications of fetuses with risk of serological screening.

Serological screening category	Number of cases	Pathogenic CNVs		DR (%)
		>10 Mb	<10 Mb	
High risk	868	23	19	4.83
Critical risk	101	1	3	3.96
Abnormal MoM	18	0	0	0

#### 3.2.3 CMA in fetuses with high risk of NIPT

We screened out 934 fetuses with positive NIPT results, including aneuploidies and segmental CNVs. A total of 395 pathogenic CNVs were detected, including 300 aneuploidies, 24 large-fragment abnormalities, 39 small-fragment abnormalities, and 32 mosaicisms. CMA increased the DR by 4.17% but failed in detecting three sex chromosome mosaicisms compared with karyotyping. Among this NIPT group, 22q11.2 micro-deletion was detected in 12 fetuses, with the PPV of 100%. 22q11.2 deletion syndrome (DS 22q11.2) is a rare disease caused by the loss of the q11.2 region of chromosome 22, also known as DiGeorge syndrome or velocardiofacial syndrome depending on the clinical presentation of each individual (Cortés-Martín et al., 2022). The common clinical manifestations are cardiac anomalies and abnormalities in the palate, thymus, or parathyroid glands.

#### 3.2.4 CMA in fetuses with advanced maternal age

We screened out 369 fetuses with isolated advanced maternal age, ranging from 35 to 47 years. CMA detected clinically significant genetic imbalances in seven fetuses, including trisomy 21 (n = 4), small CNVs (n = 2), and XXX mosaicism (n = 1). The ages of these seven pregnant women are all beyond 40 years.

#### 3.2.5 CMA in fetuses with family history

A total of 544 fetuses of mothers who had a history of a fetus or child with genetic disorders, structural anomalies, chromosomal disorders, and other unexplained abnormal were assessed. According to combined CMA and karyotyping results, clinically significant CNVs were detected in 16 fetuses, including micro-deletion (n = 7), micro-duplication (n = 3), unbalanced translocation (n = 3), trisomy 21 (n = 1), 47,XYY(n = 1), and 45, X mosaicism (n = 1).

A 276-Kb hemizygous micro-deletion of Xp21.1 was detected using CMA in a fetus of a mother who delivered a boy diagnosed with Duchenne muscular dystrophy (DMD). The Xp21.1 region contains OMIM genes including the DMD gene. Xp21 gene deletion syndrome is characterized by complex glycerol kinase deficiency, congenital adrenal hypoplasia, intellectual disability, and DMD, while the clinical features depend on the size of the deletion and the number and nature of the encompassed genes (Heide et al., 2015). We then verified the pathogenic deletion in the DMD gene, and the mother eventually chose abortion.

#### 3.2.6 CMA in fetuses with a history of adverse exposure

This group comprised 64 pregnant women who were exposed to radiation, using drugs that may be harmful to the fetus and prenatal alcohol. CMA tests only detected a large deletion in 4p16.3p15.2. Three additional small CNVs were clinically interpreted as VUS. Except for the pCNV, the remaining couples chose to continue pregnancy. They were eventually born healthy, except for one with a small duplication in 16p11.2 interpreted as VUS, which was detected as postnatal hypertonia.

#### 3.2.7 CMA in fetuses with parental chromosome anomaly

A total of 224 fetuses of mothers or fathers with chromosome-balanced or Robertsonian translocation, inversion, numerical abnormality, and mosaicism were assessed. A total of 10 clinically significant CNVs were detected, including micro-deletion (n = 4), unbalanced translocations (n = 4), 47, XXY (n = 1), and 47, XYY (n = 1). The four unbalanced translocations were all considered to result from parental balanced chromosome translocation and inversion. A 1.37-Mb deletion was detected using CMA in a fetus whose father presented with dermatofibroma. Notably, the father carries the same micro-deletion involving the NF1 gene, which is associated with neurofibroma type I. Consequently, the mother eventually chose abortion.

#### 3.2.8 CMA in fetuses with other high-risk prenatal indications

A total of 301 fetuses received invasive prenatal diagnosis for other indications in this study, including parental carriers of genetic variants, virus infection, unexplained parental phenotypic abnormalities, and other conditions considered necessary for prenatal diagnosis.

Clinically significant CNVs were detected in nine fetuses, including trisomy 21 (n = 1), 47, XXY (n = 1), and small CNVs (n = 7).

### 3.3 Variants of uncertain significance in CMA

VUS represented a broad category of CNVs which cannot be classified as either pathogenic or benign. In this study, 253 CNVs with the addition of 65 AOH were identified and clinically interpreted as VUS by CMA. The DR of VUS was 3.71%. The accreditation criteria of VUS were according to the ACMG practice guidelines, and it might be the greatest challenge of CMA in prenatal clinical application. The size threshold for analyzing CNVs was set at 400 kb for gains and 100 kb for losses (>50 markers). In addition, the classification of VUS corresponds to points between −0.89 and 0.89 using the scoring system. Interpretation of the results requires former reports in databases or previous functional studies; the lack of information made clinicians unable to predict accurate fetal prognosis or related phenotypes. Under this condition, it is helpful to evaluate specific genes that contain deletion or duplication regions. Further assessment should be provided to determine whether one parent has the same VUS detected in the fetus if necessary. Although newborn CNVs are more likely to be pathologic, the presence of the same CNVs in parents do not always rule out the possibility of fetal abnormalities. This raises a great challenge in genetic counseling. Therefore, pre-test counseling is also important to help patients understand the possibility of receiving unexpected results or results that do not have clearly established or specific clinical outcomes. With new knowledge and experience growing over time, the number of genomic regions definitively associated with disease has increased, and every CNV classified as VUS may be reclassified as benign or pathogenic. The incidence of VUS has decreased over time based on the new literature and public data sharing; a review of the same dataset on an annual basis facilitated an increase in pathogenic cases to 1.8% and a reduction in the VUS cases to 0.9% (Levy and Wapner, 2018).

The follow-up in this study showed that 83.01% pregnant woman eventually chose to give birth after variants were interpreted as VUS by CMA, except for 43 odinopoeia, 2 spontaneous abortions, and one intrauterine death owing to hydrops. Among the newborns, 22 were born with low birth weight, 4 died after birth, 1 developmental delay after birth, 1 with cleft lip, and 1 with hydrothorax. Eight were lost to follow-up. The average follow-up period for cases with unclear significance (VUS) was 1 year, and the follow-up scope covered all cases.

### 3.4 Chromosomal mosaicism in CMA

A total of 64 mosaicisms were detected in this study. A total of 49 mosaicisms were detected by karyotyping, while 61 mosaicisms were identified using CMA; however, CMA failed to detect 3 mosaicisms. Karyotype and CMA analyses can both detect aneuploid chromosomes. However, differences between the two technologies and method of processing the sample may lead to different results in the diagnosis of aneuploid chromosomes or different levels of mosaicism. Normal cells may have had a growth advantage in *in vitro* cell culture, and the abnormal cell line may have a culture disadvantage; this variable proliferation of cells with different karyotypes in culture may have contributed to the inconsistent results between CMA and karyotyping (Carey et al., 2014). CMA may have greater detection efficiency for chromosomal mosaicism because it directly uses uncultured samples, thereby avoiding culture artifacts, providing a quicker result, and yielding more accurate levels of mosaicism to make a firm diagnosis; however, according to this study, it has limitations in detecting low-proportion chromosomal mosaicism.

### 3.5 AOH in CMA

A total of 68 samples were identified as AOH by CMA, 6 of which were considered uniparental disomy (UPD). Among them, two UPDs and a large-fragment AOH occurred in chromosome 6 were identified as pathogenic or likely pathogenic. One UPD was considered paternal by following the biparental CMA test, which was associated with transient neonatal diabetes type 1. The couple eventually opted for labor induction. The other two couples refused further examination, and the baby was born with low birth weight but asymptomatic at follow-up.

## 4 Discussion

We evaluated the clinical utility of CMA for prenatal diagnosis in samples from 8,560 fetuses with various high-risk indications. Among them, 1,037 clinically significant genomic imbalances were identified. The rate of pathogenic CNVs is 12.11%, with a success rate of 99.95%. Most chromosomal abnormalities were aneuploidies, and 2.54% of the total number of pregnancies were submicroscopic CNVs. Micro-deletions were significantly more prevalent than micro-duplications. Meanwhile, the detection rate of chromosomal abnormalities by karyotyping is 9.38%, with a success rate of 99.27%. It should be noted that there were three marker chromosomes detected by karyotyping that provided negative result by CMA, and the corresponding results were verified in one of the parents eventually. These data showed that CMA has improved the pathogenic CNV DR by 2.73% compared with karyotyping, mainly based on the advantage in chromosomal submicroscopic structure. In addition, the CMA has a higher success rate than karyotyping, due to the less requirement for sample quality. CMA almost increased the DR in all high-risk indication groups, except in the history of adverse exposure group, where a large deletion was detected by CMA and karyotyping. The DR of CMA was statistically significant in indication, such as abnormal ultrasound (χ2 = 27.6, P = 0.00), risk of serological screening (χ2 = 15.74, P = 0.00), and adverse family history (χ2 = 7.34, P = 0.007). Here, it has been suggested that CMA has better detection efficiency and accuracy on diagnosing chromosomal abnormalities as a routine inspection in prenatal diagnosis.

Clinically significant genomic imbalances were detected in 6.38% of fetuses with structural anomalies by CMA. The detection rate of multiple anomalies (15.5%) using CMA exceeded that of single-system anomalies (5.46%). Among fetuses with abnormal CMA results in major structural anomalies, cardiovascular defects were the most prevalent type (26.78%), followed by urogenital defects (16.07%), both of which were relatively more frequent in fetal ultrasound structure anomalies. In single-system subgroups, the detection rate of pCNVs in hydrops fetalis (15.55%) was the highest, followed by cystic hygroma (12.5%). It is also worth mentioning that in the ultrasound soft marker group, the detection rate of pCNVs in increased NT/NF (12.03%) was also the highest among these subgroups. Some researchers defined increased thickness of NT, cystic hygroma, skin edema, and some other pathologic fetal fluid collection conditions as non-immune hydrops fetalis (NIHF) (Sparks et al., 2020; Wang et al., 2024). The related genetic disorders can be manifested by one or more abnormal fluid collection, and the types of abnormal fluid collections may change during gestation (Gedikbasi et al., 2007; Sparks et al., 2019; Baer et al., 2014). Although hydrops fetalis and cystic hygroma were classified as structural anomalies by ultrasound, NT/NF was considered ultrasound soft markers. This suggested that they may share a common pathogenic mechanism like abnormal development of the fetal lymphatic system, and the different degrees of lymphatic reflux disorder may lead to different phenotypes. Moreover, CMA should be highly recommended for prenatal diagnosis in this situation.

Thorax dysplasias are diverse, mainly including congenital diaphragmatic hernia (CDH), pulmonary sequestration, and congenital cystic adenomatoid malformation, which are commonly surgically treated. The prognosis of the fetus depends on various complications and genetic disorders. In this study, no clinically significant genomic alterations were found in 69 fetuses with thorax dysplasia, while four CNVs and one AOH for chromosome 7 were identified as VUS. Although studies showed no specific cytogenetic anomaly associated with this thorax dysplasia, chromosomal aberration was detected in 2%–33% of CDH patients, while an additional 6%–9% clinically relevant CNVs were identified using chromosome microarray (Lurie, 2003; Yu et al., 2012; Kammoun et al., 2018). Moreover, further research revealed that partial CDH were related to variants that affect known CDH causative genes (Kammoun et al., 2018). The differences in our detection results might be associated with patient selection bias and poor case number. In addition, using whole-exome sequencing (WES) as an additional testing to CMA might be a better strategy to thorax dysplasia fetuses.

The prevalence of fetal-isolated soft markers was 23.18%, while clinically significant genomic imbalances were detected in 6.29% of fetuses with isolated USMs. CMA improved the DR of pCNVs by 2.41% compared with karyotyping. The incidence of chromosomal abnormalities was significantly higher in the increased NT/NF subgroup (12.03%), which comprised 20.77% with small pCNVs.

In addition, we also evaluated two kinds of abnormal indications and three kinds of abnormal indication groups. The two kinds of abnormal indication group represents a substantial proportion of high-risk pregnant women (n = 1400, 16.35%). The incidence of chromosomal abnormalities was 22.35%, which is significantly higher than that of other groups. Special attention should be given to multiple indications for prenatal diagnosis in pre-test counseling.

The diagnostic yield of CMA is affected by the clinical indications, particular population, artificial labor, or spontaneous miscarriages. The clinical indications chosen in this study showed very different associations with chromosomal abnormalities and have a statistically significant difference; it requires great wisdom for clinicians in choosing a prenatal diagnosis strategy. Moreover, the retrospective study was limited in terms of the data acquisition due to the scarcity of detailed genotype–phenotype information of some fetuses. The fetal phenotype description might be restricted by young gestational ages, which could be more detailed and comprehensive with increasing gestational age. In addition, limitations also included the lack of long-term follow-up after birth in this retrospective study. With its application in prenatal diagnosis, CMA has revealed an increasing number of CNVs, including variants of uncertain significance. Although more molecular genetic data can be provided, the identification of VUS might cause maternal anxiety and difficulties in genetic counseling. The absence of parental data and postnatal phenotypes complicated assessments of some CNVs. The number of some subgroups was small, and the heterogeneity of samples might have an impact on deriving proper conclusions or recommendations. Finally, we found 47 cases with negative CMA results but claimed abnormal development or early death during follow-up. Unfortunately, prenatal exon sequencing was not performed at that time because of a lack of experience and technical limitations. We believe that our future studies targeting these cases, such as using WES technology, will improve the ability of prenatal diagnosis eventually. Moreover, appropriate genetic counselling regarding the advantages and limitations should be available before undergoing CMA.

## 5 Conclusion

CMA has a better diagnostic value for screening chromosomal abnormalities, especially for those pregnant women with normal karyotypes. CMA could serve as a first-tier test for different indications, especially for ultrasound abnormalities like structural anomalies and some USMs. It can facilitate prenatal diagnosis and genetic counseling.

## Data Availability

The datasets presented in this study can be found in online repositories. The names of the repository/repositories and accession number(s) can be found in the article/supplementary material.
